# Standard reporting of high-risk histopathology features in retinoblastoma

**Published:** 2018-06-03

**Authors:** Caroline Thaung, Esin Kotiloglu Karaa

**Affiliations:** 1 Consultant Ophthalmic Pathologist: Moorfields Eye Hospital, Institute of Ophthalmology, London, UK. *Teaching blog:* **https://eyepathlondon.wordpress.com/**; 2 Consultant in Paediatric Pathology: Barts Health NHS Trust, London, UK.

**Once an eye with retinoblastoma is excised, accurate histopathological staging is essential in order to determine whether the child can leave the hospital completely cured, or may need chemotherapy or radiotherapy.**

## Histopathology examination in retinoblastoma

Surgical treatment for retinoblastoma is limited to enucleation (removal of the eye) or exenteration (removal of the eye and contents of the orbit). Histopathological examination of such specimens yields information which may affect decisions about further treatment. This information should be part of the clinical information when considering future management and prognosis.

## Standard histopathological reporting of specimens

When histopathologists are dealing with cancer specimens, their reports should include standard and appropriate information items, commonly known as a dataset. In the UK, histopathologists use datasets produced by the Royal College of Pathologists. Data items are reviewed every two years and aligned with the international tumour, node, metastasis, (TNM) classification. The TNM classification for retinoblastoma has recently been updated from TNM7 to TNM8. The newest dataset for retinoblastoma from the Royal College of Pathologists (RCPath), released in early 2018, reflects the changes in the TNM classification (see p. 34).

## Histological high-risk features: rationale

The RCPath dataset for retinoblastoma records whether tumour is present in specific structures. Presence of tumour in any of these structures is considered a histological high-risk feature (HHRF). Retinoblastoma specimens with HHRFs indicate that the patient is at higher risk for metastasis (tumour spread) than a patient whose specimen does not have such features.

**PO block**

This is a slice which includes the cornea, pupil and optic disc. It is typically obtained by making two parallel slices from anterior to posterior, one either side of the limbus. The orientation of the parallel slides may be sagittal, transverse or oblique, depending on the location of the tumour and any other pathology to be sampled.

Detection of HHRFs by the histopathologist influences management decisions. The histopathologist examining the specimen should therefore specifically seek such features. The structures where tumour presence is regarded as an HHRF are:

- anterior chamber
- iris
- trabecular meshwork
- Schlemm's canal
- ciliary body
- choroid (above a certain threshhold)
- sclera
- extraocular structures
- retrolaminar optic nerve (including the cut end).

## Gross examination of enucleation and exenteration specimens

The RCPath dataset for retinoblastoma includes recommendations for macroscopic examination and sampling of globe and exenteration specimens. Even if resources are limited, it is useful to sample the cut end of the optic nerve separately (if length allows) as well as a ‘PO block’ (see panel) of the globe. If the globe is being opened fresh to sample tumour for cytogenetics, the optic nerve should be sampled first. This will avoid artefactual contamination.

The sampled optic nerve should be embedded transversely, so that a full cross-section can be evaluated. It can either be embedded on the true resection margin (surgeon's cut end) or the pathologist's cut end, as long as the orientation is known to the reporting pathologist.

**Figure 1:**
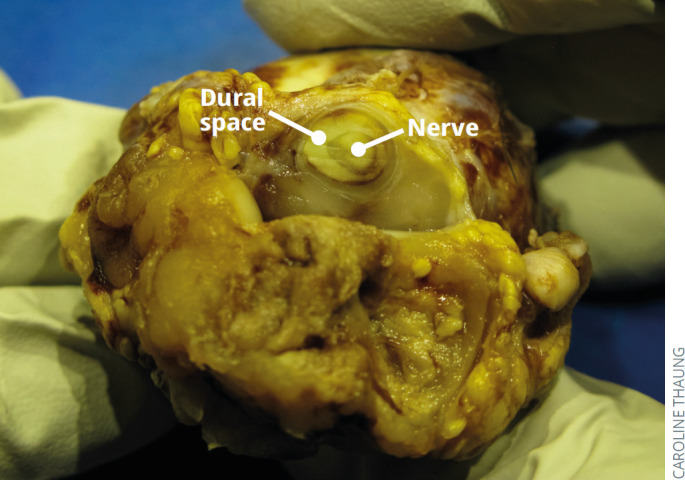
An exenteration specimen after the pathologist has cut the end of the optic nerve. Tumour partly fills the nerve and extends into the dural space.

If there is obvious extraocular extension such as an extrascleral nodule or orbital involvement in an exenteration specimen, the specimen should be sampled in such a way as to confirm this microscopically.

**Figure 2:**
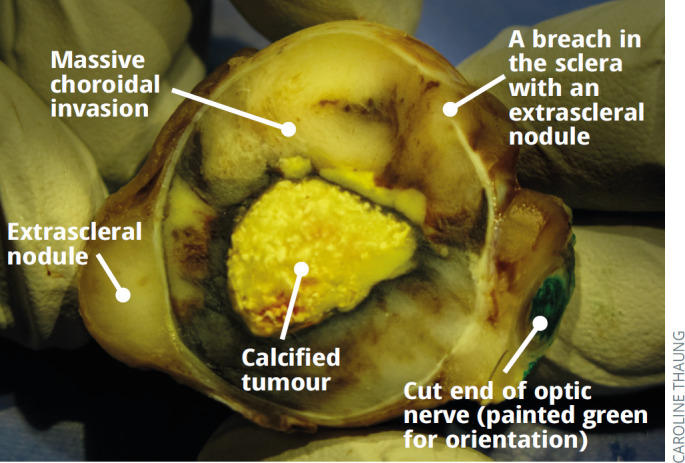
A globe with advanced retinoblastoma. There is extrascleral extension and massive choroidal involvement.

Eye structures can become distorted in advanced retinoblastoma, making microscopic examination difficult. If the globe is disorganised, PAS stain can help with orientation by highlighting Descemet's membrane, the lens capsule and Bruch's membrane.

### Anterior segment and ciliary body

**Figure 3:**
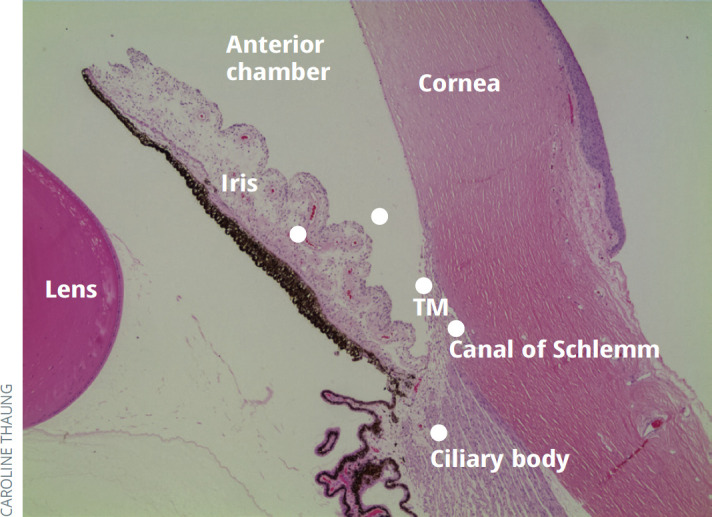
A normal anterior segment. Structures that may be involved in HHRF retinoblastoma are indicated with a white dot.

The anterior chamber is the space bounded by the cornea anteriorly, iris leaflets posteriorly and angle/trabecular meshwork peripherally. Schlemm's canal is not always easy to identify histologically, but lies adjacent to the trabecular meshwork. The ciliary body consists of the pars plicata (ciliary muscle covered internally by a double layer of ciliary epithelium) and the more posterior pars plicata.

**Figure 4:**
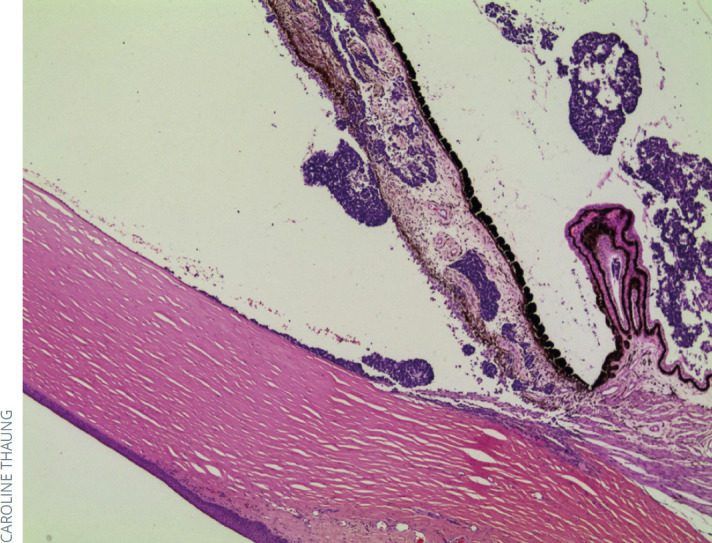
An anterior segment with HHRF retinoblastoma involving the anterior chamber, iris and trabecular meshwork.

**Figure 5:**
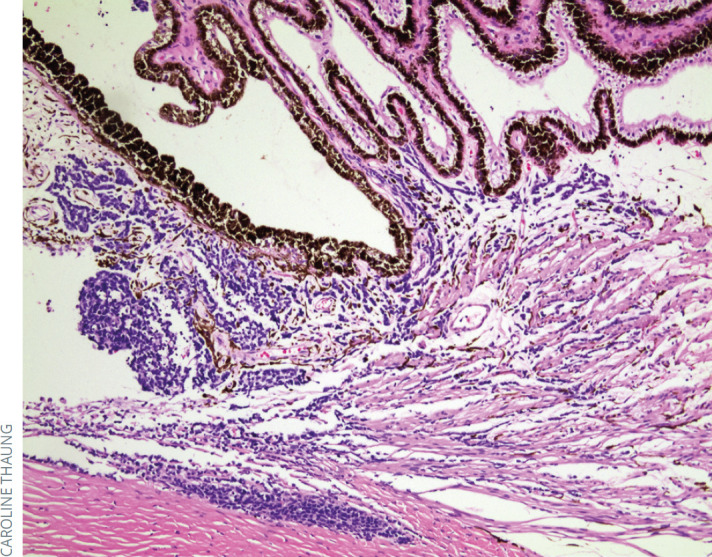
In this HHRF case, the iris, trabecular meshwork and ciliary body are invaded.

### Choroid

The choroid lies between Bruch's membrane and the sclera. It consists of a capillary network (choriocapillaris) closer to Bruch's membrane, and a vascular stroma towards the sclera. If retinoblastoma invades the choroid, tumour deposits will be seen beneath Bruch's membrane. Bruch's membrane can be difficult to see. It is highlighted with PAS stain, and the retinal pigment epithelium (RPE) may also be a useful landmark.

It is useful to comment on focal choroidal involvement (deposits measuring <3mm either singly or in aggregate), but this is not an HHRF. HHRF relating to the choroid is defined as: massive choroidal invasion (a deposit >3mm in any dimension) or multiple foci of focal choroidal involvement totalling >3mm or any full-thickness choroidal involvement (ie touching the sclera).

**Figure 6:**
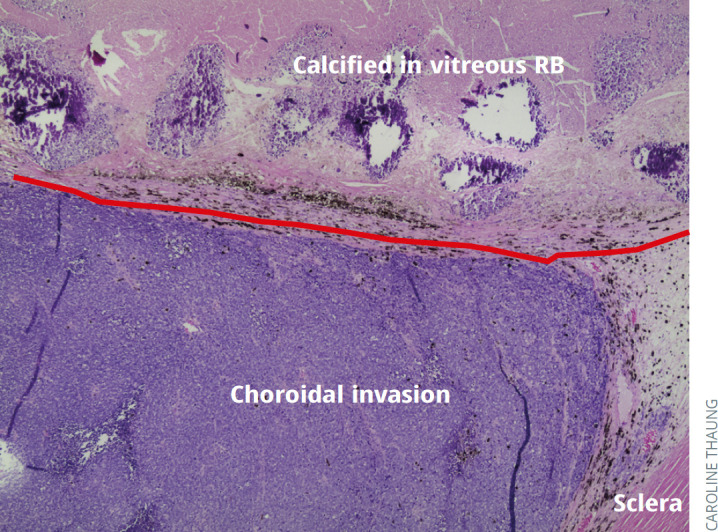
This is the case from Figure 2, demonstrating massive choroidal invasion. The intraocular structures are largely obliterated. Residual RPE (red line) indicates the approximate border of the choroid.

### Optic nerve

A transverse cross-section of the optic nerve which is free from tumour is confirmation that the (surgeon's) cut end is free from tumour. However, retrolaminar optic nerve involvement should also be assessed as it is an HHRF.

**Figure 7:**
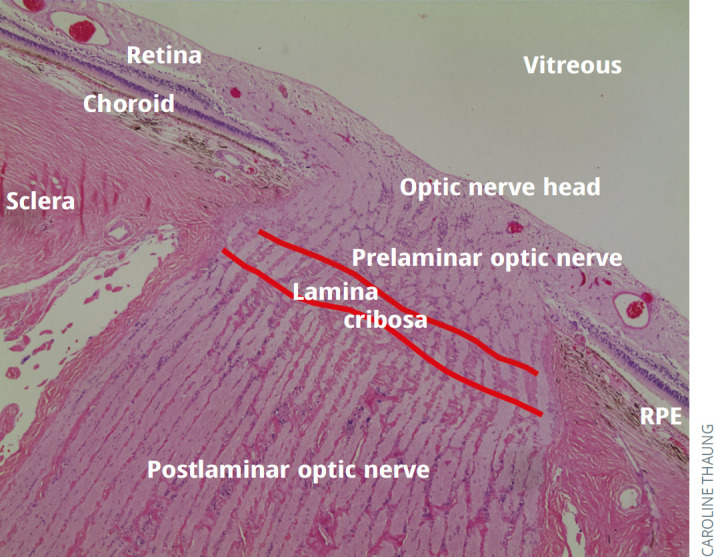
This is a normal optic nerve with the lamina cribrosa highlighted (between the red lines). Retinoblastoma invading posterior to the lamina cribrosa is an HHRF.

**Figure 8:**
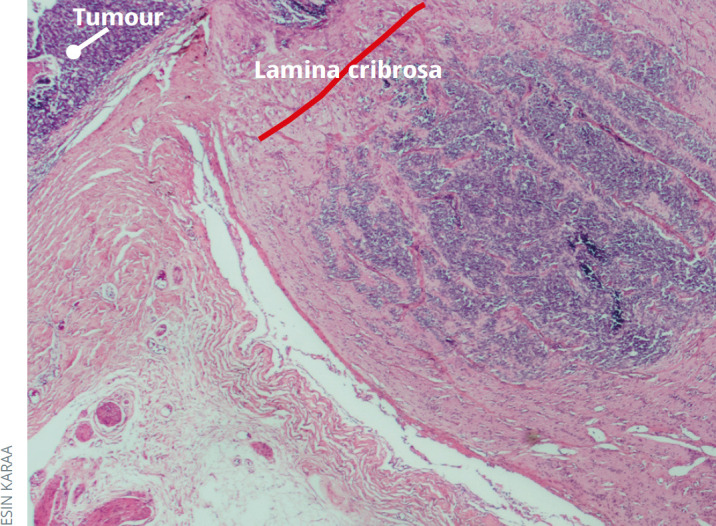
This is optic nerve with retinoblastoma invading past the lamina cribrosa (red line): postlaminar invasion.

**Figure 9:**
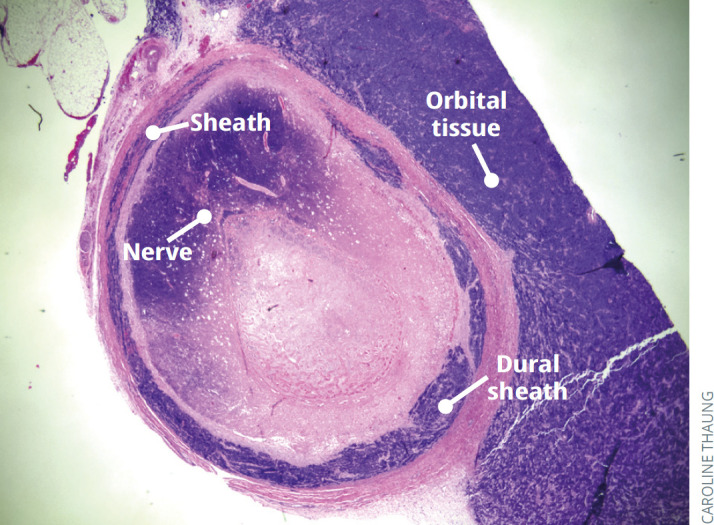
This is the optic nerve from the case illustrated in Figure 1. Retinoblastoma invades the nerve and surrounds it within the dural sheath. Tumour invades the sheath and there is also tumour in the adjacent orbital tissue.

### Sclera and extraocular

If extrascleral fibroconnective tissue is fibrotic, it is not always straightforward to discern the difference between partial thickness scleral involvement and extrascleral involvement. However, both are considered HHRFs.

**Checklist for assessing HHRF on microscopy** This is a suggested ordering of examination in order to assess whether HHRF are present, with the easiest to detect listed first.

**Table:** 

Structure	Tips on detection
Optic nerve cut end	easiest to assess in transverse section
Extraocular structures	identify the sclera
Retrolaminar optic nerve	identify the lamina cribrosa
Sclera	
Choroid (≥3mm single or aggregate, or touching sclera)	bounded by RPE/Bruchs' and sclera
Anterior chamber → iris → angle (trabecular meshwork and Schlemm's canal)	
Ciliary body	
